# SynteBase/SynteView: a tool to visualize gene order conservation in prokaryotic genomes

**DOI:** 10.1186/1471-2105-9-536

**Published:** 2008-12-16

**Authors:** Frédéric Lemoine, Bernard Labedan, Olivier Lespinet

**Affiliations:** 1 Institut de Génétique et Microbiologie, Université Paris Sud XI, CNRS UMR 8621, Bât. 400, 91405 Orsay Cedex, France; 2 Laboratoire de Recherche en Informatique, Université Paris Sud XI, CNRS UMR 8623, Bât. 490, 91405 Orsay Cedex, France

## Abstract

**Background:**

It has been repeatedly observed that gene order is rapidly lost in prokaryotic genomes. However, persistent synteny blocks are found when comparing more or less distant species. These genes that remain consistently adjacent are appealing candidates for the study of genome evolution and a more accurate definition of their functional role. Such studies require visualizing conserved synteny blocks in a large number of genomes at all taxonomic distances.

**Results:**

After comparing nearly 600 completely sequenced genomes encompassing the whole prokaryotic tree of life, the computed synteny data were assembled in a relational database, SynteBase. SynteView was designed to visualize conserved synteny blocks in a large number of genomes after choosing one of them as a reference. SynteView functions with data stored either in SynteBase or in a home-made relational database of personal data. In addition, this software can compute *on-the-fly *and display the distribution of synteny blocks which are conserved in pairs of genomes. This tool has been designed to provide a wealth of information on each positional orthologous gene, to be user-friendly and customizable. It is also possible to download sequences of genes belonging to these synteny blocks for further studies. SynteView is accessible through Java Webstart at .

**Conclusion:**

SynteBase answers queries about gene order conservation and SynteView visualizes the obtained results in a flexible and powerful way which provides a comparative overview of the conserved synteny in a large number of genomes, whatever their taxonomic distances.

## Background

As prokaryotic species diverge, their gene order is increasingly fading away, except in rare locations where a few genes retain their neighborhood. Such observations gave rise to the concept of genomic context [1-9]. Accordingly, it is assumed that a small number of genes remain adjacent either because their expressions occur at the same time, or because they encode proteins that are constituents of the same molecular machine (e.g. membrane ATPase) or involved in the same cellular function [10]. These genes that remain persistently adjacent in constantly moving genomes form synteny blocks. In a recent work [11], we have identified such synteny blocks in a large and diverse set of nearly 600 microbial genomes using a three-step process. In step one, we compared each protein encoded by a completely sequenced genome with all other available microbial proteomes in order to identify the full set of homologous proteins they share. In step two, we outlined an approach allowing the identification of *bona fide *orthologues among all recognized homologues when comparing many pairs of genomes. This second step is based on an adaptation of the method designed by Wall et al. [12] to compute the reciprocal smallest distance (RSD) that separates the homologues present in a pair of genomes. Step three allowed further research among the correctly identified orthologues to pinpoint those that belong to a minimal unit that is conserved in each pair of genomes, i.e., a pair of positional orthologous genes (POGs) that remain adjacent in each genome. Then, after extending these minimal units as far as possible, it becomes feasible to assess the relative amount and size of synteny blocks in close and distant species. Such synteny blocks are appealing candidates in the study of the mechanisms of genome evolution and in the verification of the functional annotation of neighboring genes. Accordingly, visualizing these blocks in a large number of genomes at various taxonomic distances help to study their features. In this paper, we describe how to assemble all these synteny data in a relational database (SynteBase) and we develop a tool (SynteView) to visualize all conserved synteny blocks in a large number of completely sequenced prokaryotic genomes.

## Implementation

SynteView was designed to display homology and gene context data that are organized in a relational database, SynteBase, described in detail below.

### Creating a relational database for synteny data and populating its tables with a dedicated suite of softwares and other tools

**Figure 1:**
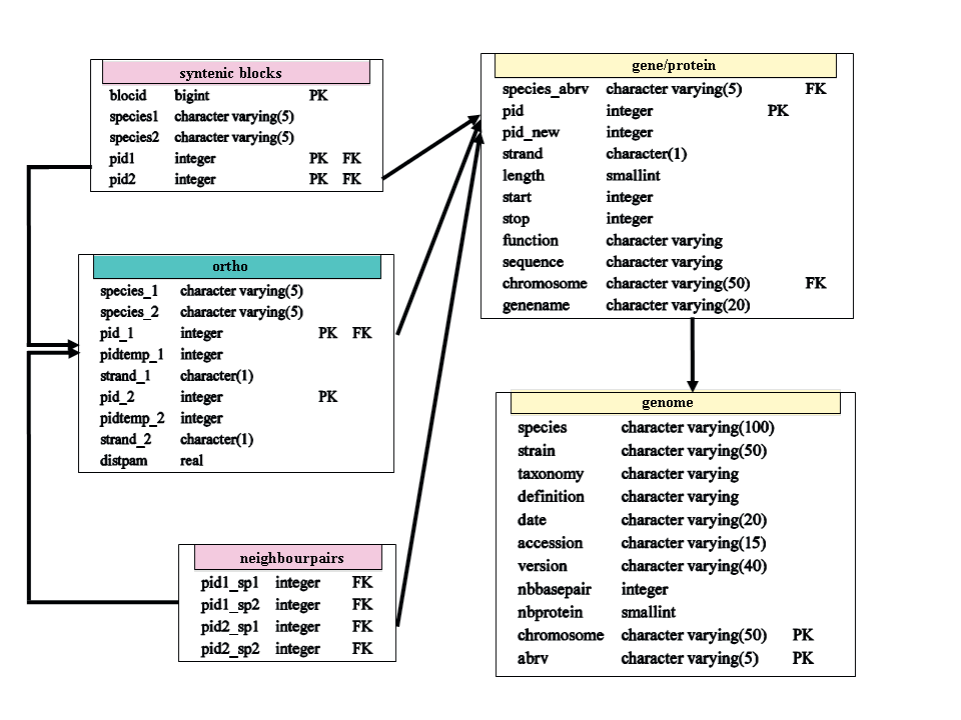
**Relational schema of SynteBase**. The SynteBase database is made up of five tables, which store information about genes/proteins, genomes, orthologous relationships, positional orthologous genes, and synteny blocks respectively. Relationships between tables are made through primary (PK) and foreign (FK) keys.

We installed PostgreSQL [13], one of the most advanced open source relational database management systems, on a Linux platform and used it to create SynteBase, which is made up of five tables (Fig. 1). The database can be further populated with home-made data using the different tools we developed (see the user guide [Additional file 1]). Alternatively, one can directly use the SynteBase version we built for our own usage (this paper and [11]).

#### Step one: searching for homologues

Raw data extracted from public genomic databanks (GenBank/EMBL/DDBJ) were organized into two tables. The *genom*e table contains information for the 598 prokaryotic genomes that were compared. The *gene/protein *table contains many features of their 1,928,135 encoded proteins, such as amino acid sequence, length, species name, location of encoding gene, etc. An exhaustive comparison of all these proteins led to the identification of all homologues. A complete suite of programs (Table 1) was used to compare each pair of proteomes using the following criteria: a pair of aligned proteins was retained as a couple of homologues if their E-values were smaller than 10^-5^, and if the alignment extended for at least 80% of the length of the shorter matching protein [11,14].

**Table 1:** A suite of programs to detect and identify synteny blocks

Step	Step designation	Tool	Reference
1	identifying homologues	protein Blast	[22]
2a	identifying orthologues by RBH	Perl script *rsd ortho*	FL, this work
2b	clustering homologues	Perl script *famtrans*	FL, this work
2c	breaking bridges	graph algorithm (Perl library)	FL, this work
2d	extracting significant clusters	MCL algorithm	[23]
3a	identifying pairs of adjacent orthologous genes	SQL query on SynteBase	FL, this work
3b	discovering synteny blocks	Perl script synblock	FL, this work

#### Step two: identifying orthologues among the collected homologues

We further adapted the Reciprocal Best Blast Hit approach [12] to analyze the Blast results obtained in the first step. The best RSD orthologous pairs were determined in each comparison of two proteomes as follows. Protein *a *encoded by genome *G*_*A *_and protein *b *encoded by genome *G*_*B *_form the best pair of orthologues if the distance separating *a *from *b *is smaller than the distance separating both *a *from any other protein encoded by *G*_*B *_and *b *from any other protein encoded by *G*_*A*_. We automated this search (Table 1, step 2a). The data obtained were used to populate the *orth*o table (Fig. 1).

#### Step three: identifying positional orthologous genes among the collected orthologues

Once populated, the first three tables were used to identify the synteny blocks. We devised a specific SQL query (see [Additional file 2]) to discover the pairs of adjacent orthologous genes (Table 1, step 3a). Then, blocks of size greater than 2 were detected by progressive accretion of blocks of size 2 which shared a common pair of orthologues (Table 1, step 3b). These computed data were entered in the *neighborpair*s and *synten*y *blocks *tables, respectively (Fig. 1).

### Architecture of SynteView

**Figure 2:**
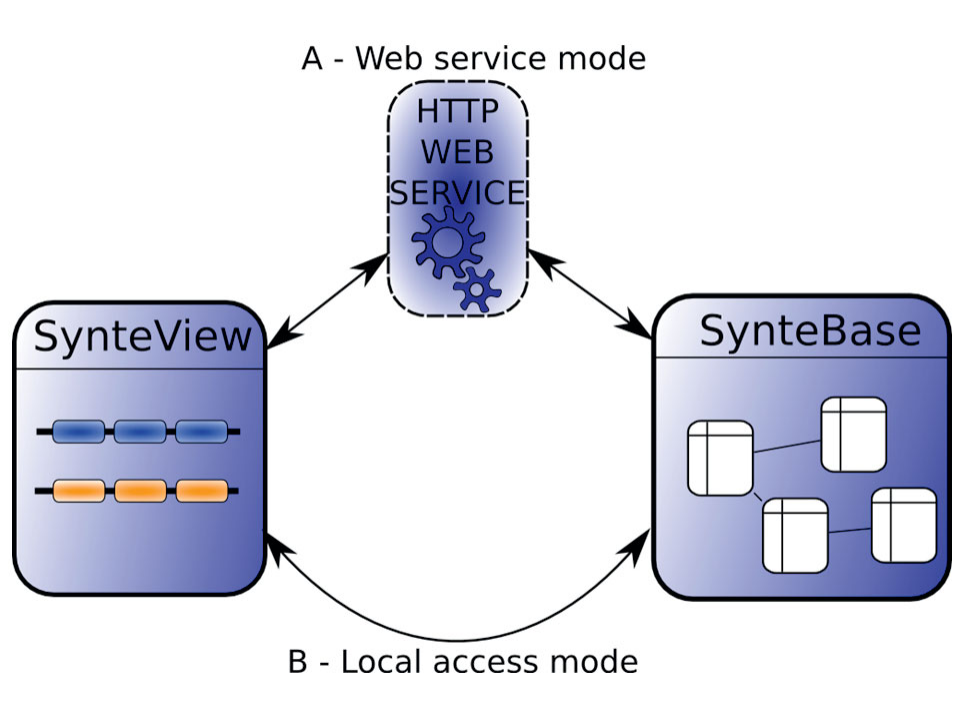
**The two ways of using SynteView**. In part A, the Web access mode connects the user to a Web service to retrieve synteny data stored in the SynteBase database . In part B, the local access mode connects the user to a local database containing the home-computed synteny data to be visualized.

To implement SynteView, we applied an object oriented programming paradigm using the Java programming language [15]. In this way, SynteView may be run either as a Java Webstart application or as a local application (Fig. 2). In both cases, SynteView can be used to query SynteBase through a web service (*web service mode*), or used to query a local synteny database (*loca acces*s *mode*). The web service mode allows the user to visualize the precomputed data that are present in our version of SynteBase. To do so, SynteView connects to the SynteView web service to retrieve synteny data present in SynteBase. The local access mode will be useful for those who wish to work online, with home-made computed data. This mode requires the local installation of the Data Base Management System PostgreSQL [13], and the creation of a committed SynteBase-like database that must be populated with home made synteny data after applying the following mandatory requirements to visualize these data. SynteView requires information on proteins (identifier, coding strand, sequence, function, and length), genomes (species name, species name abbreviation, strain name, taxonomy), and synteny blocks (identifier of the blocks, and pairs of identifiers of orthologous proteins belonging to this block). Note that it does not matter how the data are organized in the underlying local database. SynteView parameters can be set to retrieve the data it needs. However, while SynteView is independent of the name of the selected fields, their order is of importance for correct functioning. Components required to set up a local database are described in detail in the Additional file 1. Once the custom-made database has been built, SynteView can connect to it, after the settings, including connection information (server, login, etc) and all the mandatory queries have been filled out.

## Results

### Visualizing synteny data with SynteView

**Figure 3:**
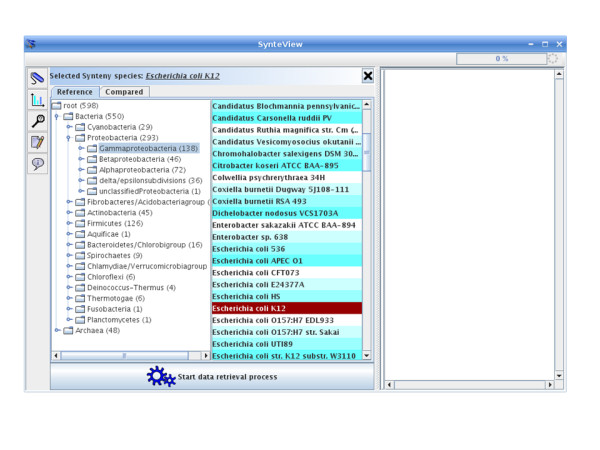
**Selecting species to be compared**. Species selection is driven by the species taxonomy. By selecting a node in the species taxonomy, the user of SynteView visualizes all the leaves descending from the selected node. It is then possible to select a species set, and finally drag and drop it into the right hand side list. The "Start data retrieval process" button starts the querying process and the visualization.

**Figure 4:**
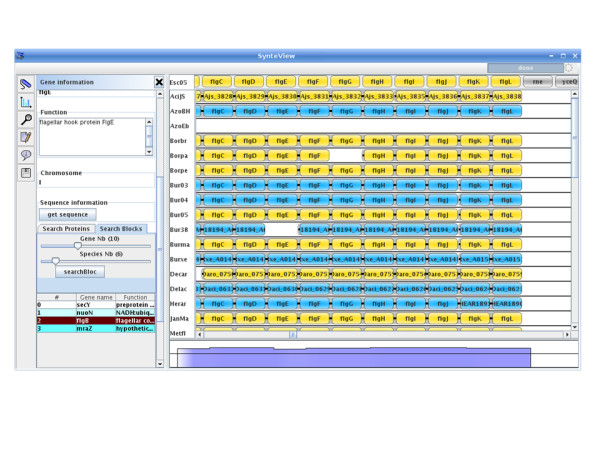
**SynteView main window**. The SynteView main window consists of a menu bar, a toolbar (on the left), a central panel which displays synteny relationships between a reference species and the compared ones, and a bottom panel, which shows the extent to which the reference species genes are conserved in blocks found in other species.

The whole set of synteny data that was stored in SynteBase was further examined using SynteView. This tool was designed to provide a wealth of information on each positional orthologous gene, to be user-friendly and customizable. For example, the user can choose the set of genomes to be studied by defining either an array of species names or a taxonomic sampling. The procedure used to visualize synteny between a reference species *s1 *and a set of species (*s2, s3, s4, s5*) is straightforward. The user first chooses a reference species, in the "select reference genome panel" by selecting nodes in the species tree (Fig. 3). Clicking on a node produces a list of all the species that are its leaves (right panel). Then, the reference species is chosen by clicking on the species name in this list. Next, the set of compared species is determined by means of the "select compared species" tab (Fig. 3). As previously noted, the user browses the taxonomic tree of prokaryotes. When the user clicks on one node of the tree (e.g. Enterobacteriales), all the descendants of this node appear in the bottom panel. To choose one or several species, a drag and drop of the selected names will move the corresponding species into the right panel. This can be repeated several times, until the required set of species is selected. When this step is accomplished, clicking on the "Start data retrieval process" button on the bottom of the panel will launch the visualization step. The speed of this process depends on the number and nature of the chosen species. Once the retrieval process is completed, all regions of each compared genome become accessible for visualization in a scrollable window using the following features as shown in Fig. 4. Each line corresponds to a genome. The first line from the top (light blue background) shows gene adjacency in the chromosome of the reference species. Dark blue (positive DNA strand) and yellow (negative strand) rectangles stand for genes belonging to a synteny block that is conserved in at least one other species. Gray rectangles are genes of this reference genome that do not have any POGs in the set of compared genomes. Respective gene names are labeled on each rectangle. The following lines contain the different species that are compared to the reference genome. SynteView automatically sorts the chosen species by their taxonomic proximity to the reference genome. For each gene of the reference genome, columns contain the orthologous genes belonging to a synteny block found to be conserved in the different analyzed genomes with their respective names. The same color code (blue or yellow) helps to discriminate the strand of their respective location on each genome. The number of genes present in a block is displayed when the cursor is run over this block. Note that synteny blocks in compared genomes are defined exclusively with respect to the gene order in the reference genome. Thus, in a SynteView window of synteny blocks, the apparent proximity in compared genomes does not imply that they are as physically close in these genomes as their POGs are in the reference genome. By opening the *Setting*s panel (to do so, click on the "settings" button in the left toolbar menu) the user accesses a Dialog box where it is possible to modify various default parameters. For example, clicking on the "Database" tab allows the user to choose the retrieval mode (database or web service). Once these various parameters have been customized, it is possible to navigate along the reference genome to estimate the density of the synteny blocks present in the other genomes. For example, and as expected, comparing *E*. *coli *with the other gammaproteobacteria reveals a rather high density of gene conservation. The bottom blue background shape portrays this rate of conservation in the compared genomes as a histogram (Fig. 4).

### Using SynteView for comparative analysis of gene context

**Figure 5:**
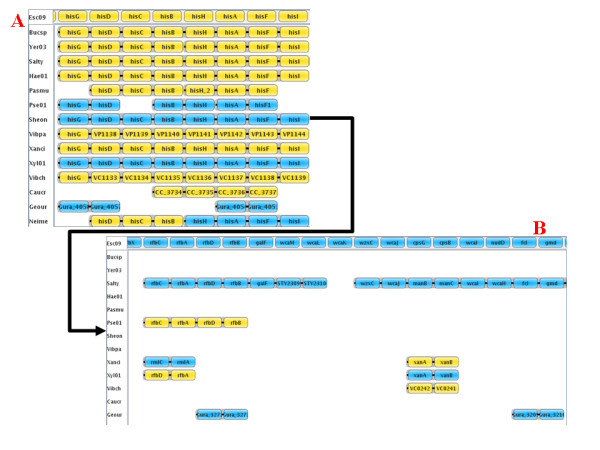
**Displaying operon conservation**. Part A shows that the order of genes belonging to the histidine operon is well conserved in this set of proteobacterial genomes. Part B shows the contrasting low conservation of the neighboring *rfb *cluster. Note that there is an interval of 12 *E*. *coli *genes (in the reference species) between gene *hisI *in panel A and gene *rfbC *in panel B.

Information about any annotated gene is immediately available by clicking on the corresponding rectangle. This opens, to the left of the window, the "gene information" panel (Fig. 4) in which, for the selected gene, its GenBank PID, its name, the species name and the replicon to which it belongs are given; the function of its product (if available), and its exact location on the chromosome are also mentioned. This information panel also contains a text field which permits simple queries such as a search for a protein function, a gene name or a PID in the analyzed genomes, as well as a search for synteny blocks containing at least *x *adjacent genes and having orthologous genes in at least *y *species. Moreover, clicking on a gene delivers complete information on its neighbors. For instance, it is possible to estimate the various levels of conservation of detected operons when comparing organisms separated by various taxonomic distances. While the operon histidine is rather well conserved in proteobacteria (Fig. 5, panel A), the neighboring clusters of genes involved in the O-specific lipopolysaccharide biosynthesis (*rfb *cluster) and the production of extracellular polysaccharide colanic acid (cluster *wca*), which are located at a short distance and on the other strand, are rapidly fragmented to a scarce number of 2–4 genes such as the *rml *genes in *Pseudomona*s *aeruginos*a (Fig. 5, panel B). In addition, clicking on the "get sequences" button in the information panel opens a dialog box. SynteView shows the sequence of the clicked gene in the first tab and that of its orthologues in the second tab in Fasta format. This further allows downloading of all these amino acid sequences for future work.

### Using SynteView for comparative analysis of multiple views

SynteView was also designed to allow complex studies by means of easy and simple operations. For example, looking at a peculiar set of species makes it possible to immediately visualize new assortments of synteny blocks. This is done simply by selecting a new reference species by clicking on a species name on the left of the display and/or by changing the list of compared genomes. Moreover, contrary to challenging tools (see Discussion below), SynteView allows global analyses of the synteny data using various points of view. Scrolling up and down the same window, one can assess the level of conservation of gene order at various taxonomic depths, the relative density of the synteny blocks along the whole genome, the relative size of the blocks, and the respective events of gene insertion/deletion in close and distant species.

### Using SynteView to quantify synteny data

Besides being a visualization tool, SynteView can display various kinds of histograms which are computed *on-the-fly*. For example, the percentage of species displaying POGs in the same synteny block in the reference species is automatically computed and displayed as a histogram (blue background shape at the bottom of the main display). It is also easy to display the distribution of the size of synteny blocks which are conserved between genomes by selecting a pair of species and clicking on the *Histogra*m button in the left toolbar. This histogram may be saved for further use by selecting the "Save as" button located in the contextual menu in the window. Table 2 summarizes the data obtained when comparing the model organism *Bacillu*s *subtili*s with various bacteria and archaea. It appears that the number of genes present in conserved synteny blocks depends on the phylogenetic (taxonomic) distance between species. Indeed, the mean size of synteny blocks is close to 3.3 genes when comparing two closely related bacteria such as the Bacillaceae *B*. *subtili*s and *Oceanobacillu*s *iheyensis*, whereas it diminishes to nearly 2 when comparing a bacterium (*B*. *subtilis*) and an archaeon (*Methanosarcin*a *acetivorans*), although these genomes encode a similar range of proteins (3000–4500). Likewise, the longest block ranges from 19 to 4 for the same species comparisons.

**Table 2:** Obtaining information on synteny blocks

species 1	species 2			synteny blocks	
				
*B. subtilis^a ^*Taxonomy	Species name	Taxonomy	Proteome size	average size	longest size
	
Bacillaceae	*Oceanobacillus iheyensis*	Bacillaceae	3500	3.6	23
Firmicutes	*Shewanella oneidensis*	Proteobacteria	4471	2.4	8
Firmicutes	*Synechocysti*s *specie*s	Cyanobacteria	3167	2.8	8
Firmicutes	*Mycobacteriu*m *tuberculosi*s	Actinobacteria	4187	2.5	11
Bacteria	*Methanosarcin*a *acetivoran*s	Archaea	4540	2.3	7

The average size (number of genes) of synteny blocks is dependent on the taxonomic distance separating a pair of genomes that have been compared at the level of their genetic context.

^a ^The size of the *B. subtilis *proteome is 4112.

## Discussion

SynteView was designed to allow fast and easy visualization of the conservation of gene adjacency in many genomes for which orthology and neighborhood data were computed and stocked in a dedicated relational database SynteBase. Our goal was to develop a flexible yet powerful tool to work directly with home-computed data obtained after comparing large and diverse sets of species. Indeed, our tool can be easily installed on any personal computer endowed with one of the main operating systems (Windows, Mac OS X or Linux). Moreover, SynteView can be customized in many aspects. In particular, it can be used with another, home-made, database in place of SynteBase. We observed that among the other tools to visualize synteny data [16-20] that have been designed to be locally installed, not one is adapted to the use of the abundant genomic data for prokaryotic species. Contrary to these previously published softwares [16-20], SynteView allows the user to compare the gene order in many different genomes in the same window. Finally, the strict relationship between SynteBase and SynteView allows their user to enlarge the study of gene order by means of specific queries on SynteBase. In addition to the visualization of synteny blocks, it is possible to obtain productive information through various requests such as "How many genes are involved in a neighbouring relationship, for each pair of genomes?"

## Conclusion

We anticipate that we will be inundated by thousands of completely sequenced genomes in the next few years [21]. Our tool SynteBase/SynteView has been designed to support such large sets of prokaryotic data. This tool will serve to quickly evaluate the conservation of gene order in newly-published genomes as soon as they have been compared to those already analyzed.

## Availability and requirements

• Project name: SynteView/SynteBase

• Project home page: 

• Operating System(s): Windows, Linux, MacOS X (Java web start)

• Programming Language: Java

• Other requirements: Java 1.5

• License: GNU GPL

• Any restrictions to use by non-academics: none

• Perl scripts: available on request

## Abbreviations

POGs: Positional Orthologous Genes; RSD: Reciprocal Smallest Distance; SQL: Structured Query Language.

## Authors' contributions

FL wrote the different programs necessary to collect all synteny data and to build up the relational database and the visualizing tool. He is responsible for the website. The three authors participated in the design of the experimental approach, the conception of the tools, and the data analysis. Together, the three authors wrote this manuscript.

## Supplementary Material

Additional file 1 **SynteView user guide**. This guide helps the user in 1) Installing SynteView, 2) Using SynteView, 3) Adapting SynteBase/SynteView to his/her own purposes. It is available at Click here for file

Additional file 2 **SQL Query computing POGs**. This file contains the SQL query for computation of POGs using *SynteBase*. The main idea is to join the *orth*o table with itself, and to take only the tuples which form a gene quadruplet where each vertical pair is made up of orthologues and each horizontal pair consists of adjacent genes in their respective genomes. Click here for file
