# Supplementary material for: SynteBase/SynteView: a tool to visualize gene order conservation in prokaryotic genomes
Source: BMC Bioinformatics. 2008 Dec 16;9:536. doi: 10.1186/1471-2105-9-536 (PMC2667195; doi:10.1186/1471-2105-9-536)
Supplement: Additional file 1 — SynteView user guide. This guide helps the user in 1) Installing SynteView, 2) Using SynteView, 3) Adapting SynteBase/SynteView to his/her own purposes. It is available at [file 1471-2105-9-536-S1.pdf]

Installing SynteView

There are two main ways using SynteView. In the Web service mode, SynteView is retrieving computed data from our database SynteBase. Alternatively, to visualize your home made synteny data from a local synteny database, you will use the local access mode.

Using SynteView Web service

There is nothing to install if your operating system contains Java (version >= 1.5),. Clicking on the Java Web Start link will launch SynteView by linking on the SynteView Web site.

Using SynteView in local access mode

1. Setting up the Database

A PostgreSQL database has to be built to contain your home-made synteny data. Your are free to design your schema but the database must contain mandatory data about

Proteins (identifier, coding strand, sequence, function, and length),

genomes (species name, species name abbreviation, strain name, taxonomy), and

synteny blocks (id, and pairs of orthologous proteins belonging to this block).

2. Configuring SynteView

Once the database is operational, several mandatory queries must be introduced that are required for visualizing gene order and conserved synteny blocks.

1. retrieving information on all genomes listed in the database:

   ```
   SELECT abbreviation,taxonomy,speciesname,nbprotein
   FROM ...
   WHERE ...
   ```

   Here, the abbreviation field stands for a short name of the species. It will be displayed in the synteny panel for visualization purpose. The taxonomy must be in the form of a list of node names, separated by a ";". The speciesname field describes the full name of the species, including the strain name if any. The nbprotein field specifies the number of proteins encoded by the genome of the species.

2. retrieving information on proteins:

   ```
   SELECT pid, chromosome, species, function, start, stop, genename, length
   FROM ...
   WHERE ...pid=%pid
   ```

   Here, the condition pid=%pid is mandatory. SynteView will replace, when necessary, %pid by the identifier of the protein under analysis. The pid field stands for the identifier of the protein, which has to be an integer. The chromosome field stands for the name of the replicon in which the gene encoding the protein under analysis is located. The species field stands for the short name of the species related to the protein. "function" is a string field representing a text description of the functional annotation of the protein. Start, stop, genename, are fields describing properties of the gene encoding the protein under analysis, respectively, and length is the amino acids length of the protein.

3. retrieving information on a genome:

```
SELECT pid, genename, strand, start, chromosome, function
FROM ...
WHERE ... abbreviation='%spabrv'
```

The condition abbreviation='%spabrv' is mandatory. SynteView will replace %spabrv by the corresponding species abbreviation when necessary. The definition of the different fields is as previously described.

4. retrieving information on syntenic blocks:

```
SELECT blockid, pid1, strand1, start1, stop1, genename1, pid2, strand2,
genename2
FROM ...
WHERE ... abbreviation1= %spabrv and abbreviation2 in (%sptab)
```

Here, the pid1, strand1, start1, and abbreviation1 stand for information about the reference genome "%spabrv", and the pid2, etc. for information about the compared genomes "%sptab".

Instruction manual

Initializing SynteView before use

To use the local acces mode, please fill in the database connection fields on the settings panel as follows: database server name, port, login, and password. Note that these fields are filled in by default in the web service mode.

Tool bar

All the actions that can be performed with SynteView are accessible via the toolbar on the left hand side of the graphical user interface. Various buttons allow to display the following panels:

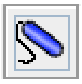	Choosing the reference species and the set of compared species
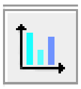	Building histograms representing the size distribution of the synteny blocks
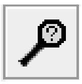	Showing and searching proteins information
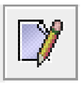	Opening the preferences dialog
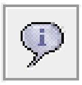	Opening the about panel
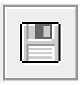	Saving synteny data as CSV

The buttons accessible on the ToolBar

The central panel

It displays the strict conservation of gene order, in comparing to a reference species (first line), chosen by the user, all other available species which are automatically sorted by taxonomy. Each gene present in the genome of the reference species is depicted by a rectangle with a color code: blue (positive strand) or yellow (negative strand). Grey rectangles are genes without orthologs belonging to a synteny block in another species.

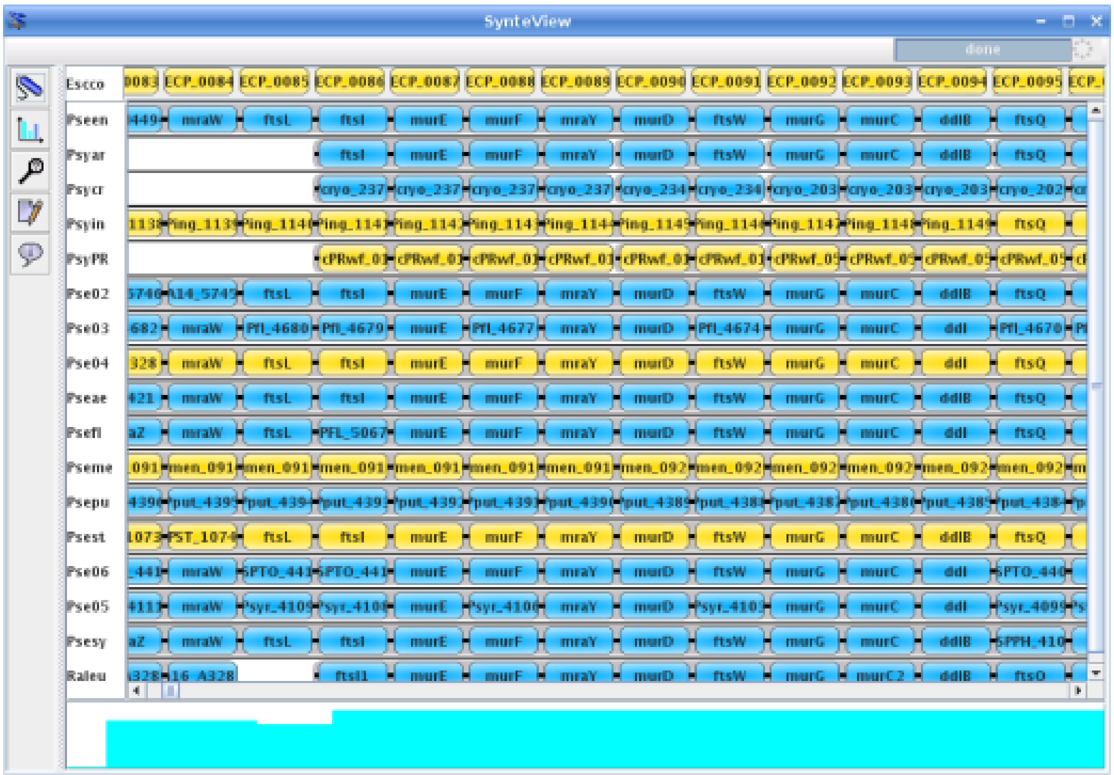

Conserved gene neighbouring in the compared species are exhibited in the next lines using the same colour code. Please remember that blocks which appear as adjacent in compared genomes are not necessarily physically neighbouring in the actual genome.

Choosing the reference species and the set of compared species

The figure below shows the species choosing panel. It contains two tabs. The first one is dedicated to choose the reference species, and the second one to choose the set of compared species. All these process are guided with the species taxonomy (according to the NCBI).

Selecting the reference species

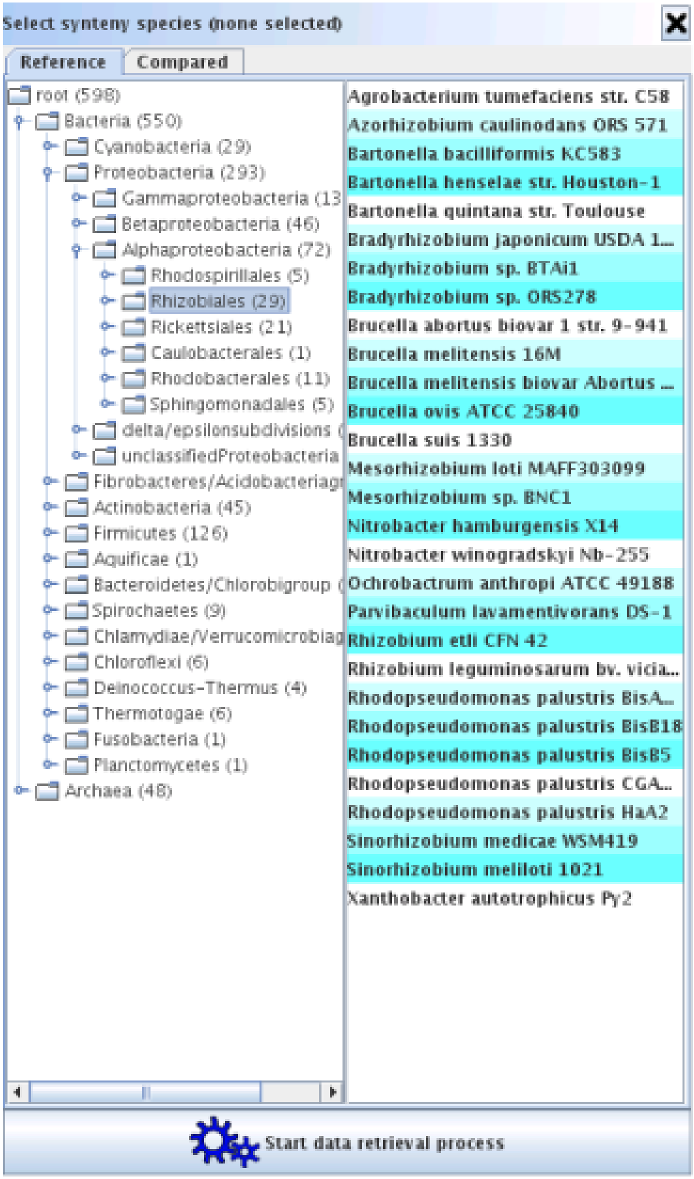

When browsing the taxonomy, please select a particular taxon (for instance, Rhizobiales). Once selected (on the left hand side of the panel), all the species contained in this taxon are displayed on the right hand side of the panel. Clicking on a particular species will select it as a reference species.

Selecting the compared species

When browsing the species tree and selecting a particular taxon as described above, its species are displayed and can be drag and drop to the right panel. This can be repeated. For instance, in the screenshot below, several cyanobacteria have been selected and it is now possible to add various archaeal species.

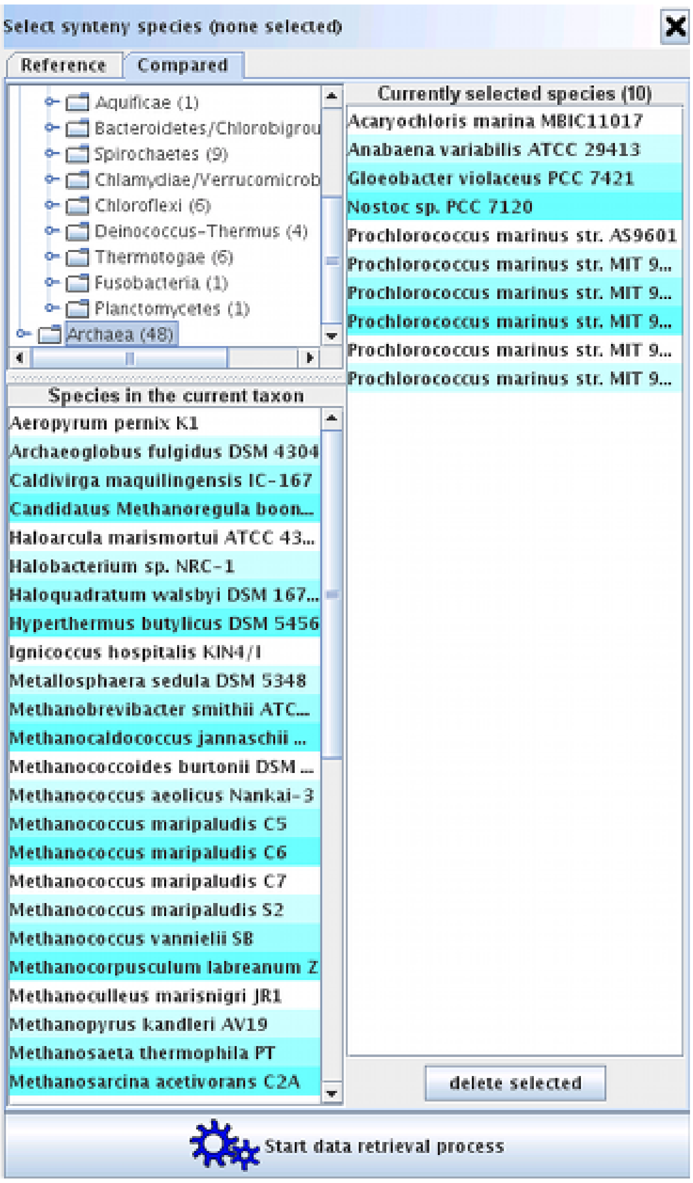

Gene information Panel

Clicking on either a specific gene or on the button (3) opens an information panel (located on the left hand side) that shows several data such as its gi number (identifier), the name of its species (Species), its location (Chromosome, Start, Stop), its name (Gene name), and its function (Function) as described in GenBank.

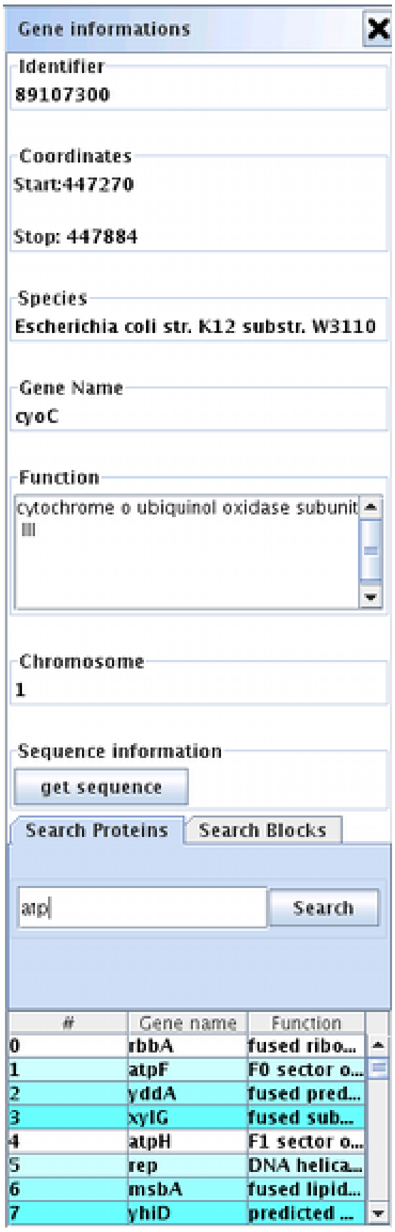

Moreover, the frame "Sequence information" allows getting the amino acid sequence (in Fasta format) encoded by the selected gene, as well as the amino acid sequence of its orthologues in other species.

In the "Search proteins" tab, a text field allows the user to query the database. For example, searching "atp" will open a window listing all related genes. Clicking on these genes allow to look at their conservation in the selected species.

Finally, in the "Search blocks" tab, one can search synteny blocks containing at least x adjacent genes and having orthologous genes in at least y species. x and y can be parametrized with sliders.

Histogram

In the bottom, a histogram shows for each gene the percentage of species in which this gene is belonging to a synteny block found in the reference species. Accordingly, one can see the width of large intervals where synteny is well conserved such as in operons for instance.

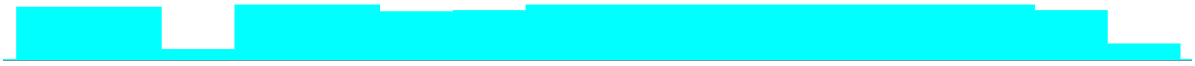

Statistical analysis

Besides visualizing gene order, SynteView can compute the size distribution of synteny blocks. Clicking the menu button (2) opens a new panel allowing choosing a species to be compared with the reference species. Once this species is chosen, the distribution of the size of synteny blocks between both species is automatically computed and shown as a histogram.

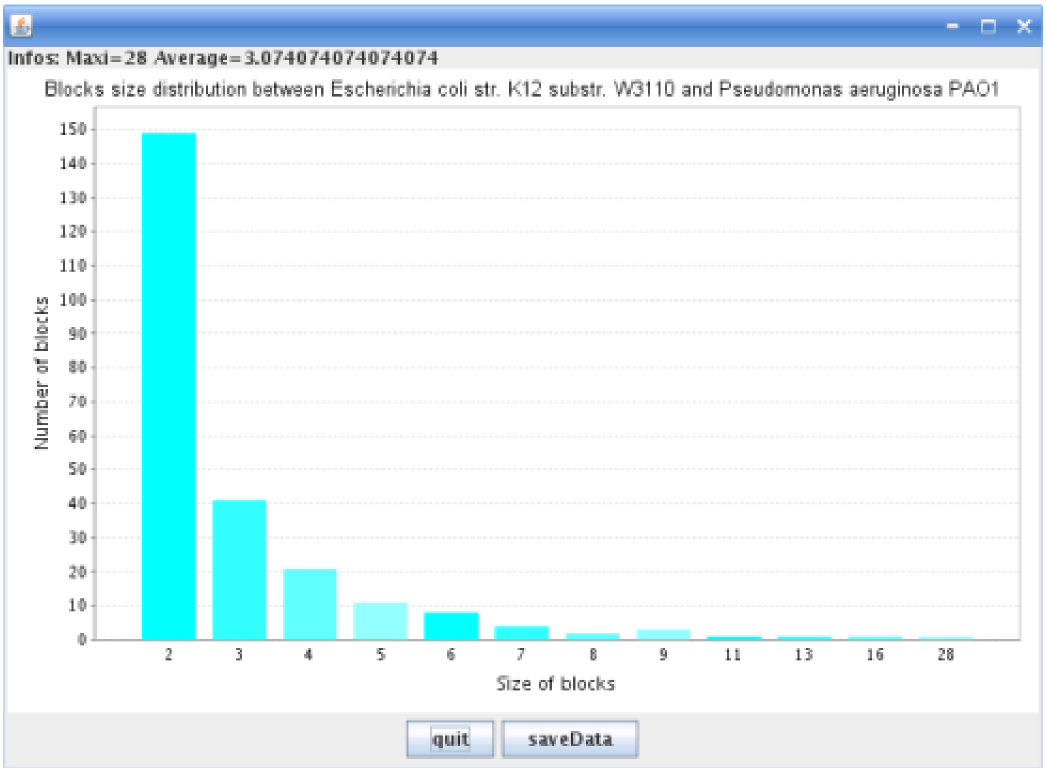

The example above shows the size distribution of synteny blocks that are conserved between the two gammaproteobacteria *Escherichia coli* and *Pseudomonas aeruginosa*.

Settings

First tab: Database access

This tab gathers several settings and the available information is dependent on the mode on which you want to use SynteView.

- If you use the Web service mode, SynteView needs the URLs from which it can retrieve the data. These URLs are filled by default.
- In the local acces mode, SynteView needs not only the database access information, but also the queries that are described in the section "Initializing SynteView before use".

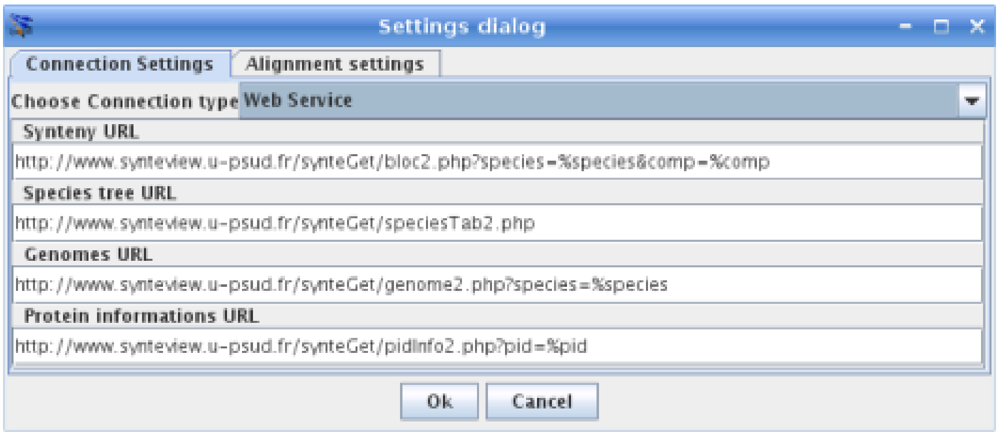
Settings dialog
Connection Settings
Alignment settings
Choose Connection type
Web Service
Synteny URL
http://www.synteview.u-psud.fr/synteGet/bloc2.php?species=%species&comp=%comp
Species tree URL
http://www.synteview.u-psud.fr/synteGet/speciesTab2.php
Genomes URL
http://www.synteview.u-psud.fr/synteGet/genome2.php?species=%species
Protein informations URL
http://www.synteview.u-psud.fr/synteGet/pidInfo2.php?pid=%pid
Ok
Cancel

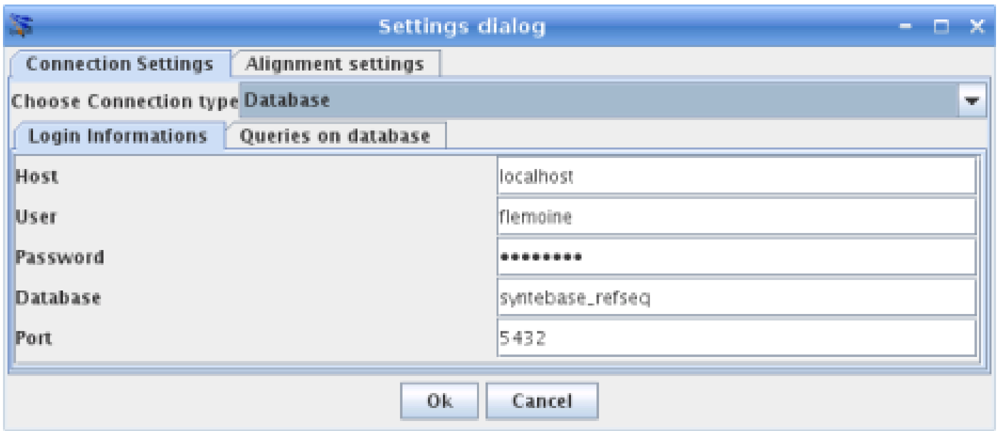
Settings dialog
Connection Settings
Alignment settings
Choose Connection type
Database
Login Informations
Queries on database
Host
localhost
User
flemoine
Password
Database
syntebase_refseq
Port
5432
Ok
Cancel
