# Supplementary material for: SynteBase/SynteView: a tool to visualize gene order conservation in prokaryotic genomes
Source: BMC Bioinformatics. 2008 Dec 16;9:536. doi: 10.1186/1471-2105-9-536 (PMC2667195; doi:10.1186/1471-2105-9-536)
Supplement: Additional file 2 — SQL Query computing POGs. This file contains the SQL query for computation of POGs using SynteBase. The main idea is to join the ortho table with itself, and to take only the tuples which form a gene quadruplet where each vertical pair is made up of orthologues and each horizontal pair consists of adjacent genes in their respective genomes. [file 1471-2105-9-536-S2.pdf]

***SQL query allowing the computation of the POGs using SynteBase*.**

```
select b1.pid_1 as pid1_sp1,
b1.pid_2 as pid1_sp2,
b2.pid_1 as pid2_sp1,
b2.pid_2 as pid2_sp2
from ortho as b1,
ortho as b2,
protein as p1,
protein as p2,
protein as p3,
protein as p4
where p1.pid = b1.pid_1
AND p2.pid = b1.pid_2
AND p3.pid = b2.pid_1
AND p4.pid = b2.pid_2
AND p3.species_abrv=p1.species_abrv
and p4.species_abrv =p2.species_abrv
and p1.pid_new = (p3.pid_new+1)
and (p2.pid_new = (p4.pid_new+1) OR p2.pid_new = (p4.pid_new-1))
and p1.strand = p3.strand  and p2.strand = p4.strand;
```
